# Machine Learning Applications for Wearable Sensor Data in Tennis and Padel: A Systematic Review of Movement Recognition, Performance Monitoring, and Emerging Injury-Related Applications

**DOI:** 10.3390/jfmk11040391

**Published:** 2026-09-29

**Authors:** Lucrezia Moggio, Antonio Ammendolia, Alessandro de Sire, Nicola Marotta, Andrea Demeco, Melania Mercurio, Olimpio Galasso, Umile Giuseppe Longo, Michele Mercurio

**Affiliations:** 1 Rehabilitation Unit, Ospedale degli Infermi, 13900 Biella, Italy; 2 Research Center on Musculoskeletal Health, MusculoSkeletalHealth@UMG, Magna Graecia University, 88100 Catanzaro, Italy; 3 Physical and Rehabilitative Medicine, Department of Medical and Surgical Sciences, University of Catanzaro “Magna Graecia”, 88100 Catanzaro, Italy; 4 Department of Life Sciences, Health, and Health Professions, Link Campus University, 00165 Rome, Italy; 5 Unit of Audiology, Phoniatrics and Vestibology, Regional Centre for Cochlear Implants and ENT Diseases, Department of Experimental and Clinical Medicine, “Magna Graecia” University, 88100 Catanzaro, Italy; 6 Department of Medicine, Surgery and Dentistry, University of Salerno, 84081 Baronissi, Italy; 7 Fondazione Policlinico Universitario Campus Bio-Medico, 00128 Rome, Italy; 8 Research Unit of Orthopedic and Trauma Surgery, Department of Medicine and Surgery, Università Campus Bio-Medico di Roma, 00128 Rome, Italy; 9 Department of Orthopedic and Trauma Surgery, “Magna Graecia” University, “Renato Dulbecco” University Hospital, 88100 Catanzaro, Italy

**Keywords:** machine learning, wearable sensor data, tennis, padel, movement analysis, rehabilitation, sport rehabilitation

## Abstract

**Background:** Wearable sensors combined with machine learning (ML) offer new opportunities for movement analysis and performance monitoring in tennis and padel. However, the current evidence and potential applications remain unclear. This systematic review aimed to summarize the applications of ML to wearable sensor data in tennis and padel, focusing on movement recognition, performance monitoring, workload and fatigue assessment, injury-related applications, and rehabilitation. **Methods:** PubMed and Scopus were searched from inception to 1 August 2026. Studies were eligible if they investigated tennis or padel, used wearable sensors, and applied ML or deep learning methods. Study selection and data extraction were independently performed by two reviewers. Risk of bias was assessed across domains related to participants, predictors, outcomes, and analysis/validation. Due to substantial heterogeneity, a qualitative synthesis was performed. **Results:** Of 393 records identified, 16 studies were included. Fourteen investigated tennis, one padel, and one included tennis data within a multisport study. IMU-based systems were the most frequently used technology. Most studies focused on movement and stroke recognition, while fewer addressed performance, workload, fatigue, or injury-related applications. Reported model performance was generally high, but validation strategies were heterogeneous, and participant-independent or external validation was uncommon. Nine studies presented some concerns, and seven were classified as having high risk of bias. Evidence directly addressing rehabilitation and return-to-sport was particularly limited. **Conclusions:** ML applied to wearable sensor data shows promising potential for movement recognition and performance assessment in tennis and padel. However, methodological limitations, small and selected cohorts, and limited external validation restrict current generalizability and practical translation. Future research should prioritize larger and more diverse cohorts, subject-independent and external validation, and prospective studies addressing injury prevention, rehabilitation, and return-to-sport.

## 1. Introduction

Tennis and padel are highly dynamic racket sports characterized by repeated high-intensity actions, rapid changes in direction, complex stroke patterns, and substantial mechanical and physiological demands [1,2]. Quantitative monitoring of these demands may provide valuable information for performance optimization, training prescription, fatigue management, and injury prevention [3,4,5]. However, conventional approaches to performance and movement assessment often rely on laboratory-based measurements, manual observation, or intermittent evaluations, which may limit the ability to continuously characterize athletes during training and competition [6]. Wearable sensor technologies have emerged as promising tools for overcoming some of these limitations [6]. Inertial measurement units (IMUs), accelerometers, gyroscopes, pressure sensors, and multimodal wearable systems can capture movement, biomechanical, physiological, and performance-related signals in relatively non-invasive and ecologically valid conditions [7]. Their use enables the collection of large amounts of data during real-world sporting activities, potentially providing continuous information on movement patterns, technical execution, external load, and athlete status [8]. Previous research has highlighted the growing potential of wearable sensors for sport performance evaluation, as well as for the assessment and monitoring of sport-related musculoskeletal injuries and recovery [6,9,10]. The increasing volume, complexity, and temporal nature of wearable-derived data, however, also create a need for analytical approaches capable of identifying patterns that may not be readily captured using conventional statistical methods [9]. Machine learning (ML) and deep learning techniques can process multidimensional sensor data and have been increasingly applied to sport-specific movement recognition, activity classification, performance assessment, workload monitoring, and injury-related prediction [5,11,12,13,14]. Systematic reviews across sports have demonstrated the potential of ML-based approaches, particularly for automated recognition of sport-specific movements, while also highlighting substantial variability in data preprocessing, model development, and validation strategies [11,15,16]. In racket sports, the combination of wearable sensors and ML may therefore provide a promising framework for moving from raw sensor signals toward automated and individualized information on movement, technical performance, workload, fatigue, and injury-related characteristics [3,17,18]. Nevertheless, the available evidence appears fragmented across different sensor technologies, anatomical locations, analytical tasks, ML algorithms, and validation strategies [3,15,19]. Moreover, it remains unclear how extensively these approaches have been investigated specifically in tennis and padel and whether current evidence extends beyond movement recognition toward clinically and practically relevant applications such as injury prevention, rehabilitation, and return-to-sport. Recent literature on wearable technologies in sports medicine has emphasized that the ability to collect biomechanical data does not necessarily translate into demonstrated clinical utility or validated injury-prevention applications [9,12]. Similarly, systematic reviews of ML-based injury prediction have highlighted substantial methodological heterogeneity and the need for more rigorous validation before these models can be reliably translated into clinical or applied settings [12,20].

Previous reviews have examined the use of ML in tennis and the broader application of wearable technologies in sport. A recent systematic review of ML applications in tennis primarily addressed performance-related domains, including psychological-state monitoring, talent identification, match-outcome prediction, tactical analysis, and training or injury-related applications, but did not specifically focus on the integration of wearable sensor data with ML across different analytical and clinical applications [21]. More recent work has also systematically reviewed wearable-sensor applications for performance assessment in tennis, highlighting the growing use of inertial sensors for stroke detection, workload characterization, and real-time monitoring, while emphasizing the need for further validation and broader athlete representation [22]. However, these reviews have either focused predominantly on tennis performance or considered wearable technologies and ML separately. To our knowledge, a systematic synthesis specifically examining how wearable sensor data are combined with ML methods across movement recognition, technical performance, workload and fatigue monitoring, and injury-related applications in tennis and padel has been lacking.

Tennis and padel share the use of wearable-based movement and performance monitoring approaches and the common methodological challenges associated with applying ML to sport-specific sensor data. At the same time, the two sports differ substantially in court geometry, wall interactions, movement demands, rally structure, and tactical requirements. Therefore, this systematic review aimed to summarize the current applications of ML methods to wearable sensor data in tennis and padel, with particular focus on movement and stroke recognition, performance monitoring, workload and fatigue assessment, and injury-related applications. In addition, we aimed to characterize the wearable technologies and variables employed, as well as the ML approaches and validation strategies adopted, and to identify current evidence gaps regarding injury prevention, rehabilitation, and return-to-sport applications. By considering the two sports within the same methodological framework while retaining sport-specific findings, the present review aimed to identify both common applications and evidence gaps that may be specific to tennis or padel. Thus, the novelty of the present review lies in integrating the technological and analytical dimensions of wearable-based ML with an assessment of validation and translational relevance across two racket sports, while explicitly distinguishing established movement-recognition applications from emerging injury-related applications and currently unsupported clinical applications.

## 2. Materials and Methods

### 2.1. Study Design and Search Strategy

A systematic review of the literature was conducted to investigate current applications of ML methods to wearable sensor data in tennis and padel. PubMed and Scopus databases were systematically searched from their inception to 1 August 2026. The searches were conducted on 20 August 2026, using predefined terms related to the sport, wearable technologies, and ML/artificial intelligence. The database-specific search strategies are reported in Table 1, while the complete search strings as executed, together with the search date, search period, and filters applied, are provided in Supplementary Table S1 to ensure reproducibility. PubMed was selected for its comprehensive coverage of biomedical and health-related literature, whereas Scopus was selected for its broader multidisciplinary coverage, including research relevant to sports science, technology, engineering, and data science. The complementary coverage of these databases has been previously described in the literature [23]. No language filter was applied during the database searches; English-language eligibility was assessed during the screening process. The review process was conducted according to the principles of the Preferred Reporting Items for Systematic Reviews and Meta-Analyses (PRISMA) 2020 statement. The systematic review protocol was registered in PROSPERO (CRD420261512054).

### 2.2. Eligibility Criteria

After duplicate removal, two reviewers independently screened all papers for eligibility. In case of disagreement, a consultation with a third reviewer allowed consensus. Articles were considered eligible if they: investigated tennis or padel; used one or more wearable sensors to collect movement, biomechanical, physiological, or performance-related data; applied a machine learning or deep learning method to the collected data; reported an application related to movement recognition, stroke classification, performance monitoring, workload monitoring, fatigue detection, injury-related assessment, or rehabilitation; and were published as peer-reviewed full-text articles.

For the purposes of this review, wearable technology was defined as a body-worn or body-integrated sensing system capable of acquiring quantitative data during tennis or padel activity. Eligible technologies included inertial measurement units (IMUs) and their constituent accelerometers and gyroscopes, pressure- and force-related sensors, flexible resistive or piezoresistive sensors, intelligent insoles and sensor arrays, and wrist- or cervical-worn sensing systems. Both single-sensor and multisensor configurations were eligible, including bilateral IMU configurations and multimodal systems combining IMU-derived signals with surface electromyography (sEMG) or other physiological signals. Eligibility was based on the use of wearable-derived data as an input to the analytical or machine-learning approach, irrespective of the specific device or manufacturer.

ML was defined as a data-driven computational approach in which an algorithm learns patterns or relationships from the acquired data to perform a classification, recognition, prediction, estimation, clustering, or related analytical task. Both conventional ML and deep-learning approaches were eligible. For data extraction and synthesis, the reported approaches were categorized according to their principal methodological framework. Conventional ML approaches included support vector machines (SVM), k-nearest neighbours (KNN), decision trees, random forests and other ensemble methods, Gaussian mixture modelling, linear discriminant analysis, clustering approaches, and eigenvalue-based classification. Deep-learning approaches included deep neural networks, convolutional neural networks (CNNs), long short-term memory (LSTM) networks, spatio-temporal graph convolutional networks, and Transformer-based models.

Studies were considered eligible when wearable sensor data were directly incorporated into the ML workflow. Signal-processing or feature-extraction procedures performed before ML were not considered independent ML methods. Conversely, studies combining multiple algorithms or sensing modalities were retained and classified according to the principal or combined ML framework reported by the authors.

Conference papers, book chapters, studies not involving wearable sensor data, studies not involving ML-based analysis, and studies unrelated to tennis or padel were excluded. Also, we excluded studies written in a language different from English.

### 2.3. Study Selection

Study selection was performed in two sequential stages. Study selection was performed in two sequential stages. First, titles and abstracts were independently screened by two reviewers according to the predefined eligibility criteria. Records were excluded at this stage when the available information clearly indicated that they did not concern tennis or padel, did not involve wearable sensor data, did not apply machine learning or deep learning methods, or did not address one of the predefined applications of interest. Clearly ineligible publication types or articles not meeting the predefined language criterion were also excluded when this could be established from the title or abstract. When eligibility could not be determined from the title and abstract alone, the record was retained for full-text assessment.

Potentially relevant records were subsequently assessed at full-text level. Reasons for exclusion were recorded during the full-text assessment. The main reasons for exclusion were the absence of an appropriate wearable-sensor/ML methodology, an unsuitable study design or publication type, or failure to meet the predefined topical eligibility criteria. In case of disagreement at either stage, a consultation with a third reviewer allowed consensus.

Reasons for exclusion were recorded during the full-text assessment. The main reasons for exclusion were the absence of an appropriate wearable-sensor/ML methodology or an unsuitable study design/publication type.

### 2.4. Data Extraction

Study selection was performed in two sequential stages: title/abstract screening followed by full-text assessment. Reference management and screening were performed using Zotero. Two reviewers independently screened all records at both stages according to the predefined eligibility criteria. Reviewers performed their assessments independently and subsequently compared their decisions. When the two reviewers disagreed on the eligibility of a record, the article was assessed independently by a third reviewer. Disagreements were resolved on the basis of the third reviewer’s assessment. No formal inter-reviewer agreement statistic was calculated.

At the title/abstract stage, records that did not meet the predefined eligibility criteria were excluded. Potentially eligible studies were retrieved for full-text assessment. At the full-text stage, 14 articles were excluded: five were conference papers, two were book chapters, four did not meet the predefined eligibility criteria regarding the sensing technology, and three did not meet the predefined study-type criteria. The complete study-selection process and exclusion reasons are reported in the PRISMA 2020 flow diagram (Figure 1).

### 2.5. Risk-of-Bias Assessment

Because of the methodological heterogeneity of the included literature, risk of bias was assessed across domains addressing participants/data sources, predictor or sensor measurement, outcome/reference standard, and analysis/validation. For the predictor or sensor-measurement domain, particular attention was given, where information was available, to sensor type and placement, measurement and acquisition procedures, calibration, sampling characteristics, signal quality, and potential sources of measurement error such as motion artifacts, sensor misalignment, drift, limited measurement range or saturation, and synchronization of multiple sensor streams. For studies involving prediction or classification models, the assessment was conceptually aligned with the domains of PROBAST+AI [23], which specifically addresses quality, risk of bias, and applicability of prediction models developed using regression or artificial intelligence methods. PROBAST+AI was selected because it provides a structured framework for evaluating studies that develop or evaluate prediction or classification algorithms, including approaches based on artificial intelligence and machine learning. The framework considers four key domains relevant to data-driven modelling: participants and data sources, predictors, outcome, and analysis. Although PROBAST+AI was originally developed for prediction modelling in healthcare, its domains address methodological aspects that are also relevant to wearable-sensor-based ML studies, particularly participant/data selection, measurement of input variables, definition of the target outcome, and model development and evaluation procedures.

Because the studies included in this review involved wearable-sensor data collected during tennis or padel activities rather than clinical prediction models, the signalling questions were adapted to the sports and wearable-sensor context without changing their underlying methodological constructs. The adapted tool is provided in Supplementary Table S2.

For model development, 16 signalling questions were considered across the four domains, whereas model evaluation was assessed using 18 signalling questions. Signalling questions were answered as yes, probably yes, probably no, no, no information, or not applicable when appropriate. Domain-level judgements were classified as low, high, or unclear. Applicability was additionally assessed with respect to participants/data sources, predictors, and outcomes, considering the extent to which the reported study populations, wearable-sensor configurations, preprocessing procedures, and classification targets corresponded to the intended application addressed by this review.

For the present review, particular attention was given to the independence of observations and to potential information leakage between training and evaluation datasets. When multiple recordings or stroke events were obtained from the same athlete, the evaluation strategy was considered participant-independent only when data from the athlete used for testing were not available during model development. Random splitting or cross-validation performed at the stroke or sample level was therefore not considered equivalent to participant-independent validation. Particular attention was also paid to the adequacy of the number of independent participants, handling of class imbalance, preprocessing procedures, separation of training and test data, resampling procedures, and the appropriateness of reported performance metrics.

Two reviewers independently assessed the methodological quality/risk of bias of the included studies using the adapted tool. Disagreements were discussed and resolved by consensus; when necessary, a third reviewer adjudicated. The study-level assessments are reported in Supplementary Table S3.

### 2.6. Data Synthesis

Because of the substantial heterogeneity in study populations, wearable technologies, sensor configurations, ML algorithms, target outcomes, and validation procedures, a qualitative synthesis was performed rather than a meta-analysis. The included studies were grouped according to their principal application: movement and activity recognition; stroke and technical-action classification; performance and skill assessment; workload and external-load monitoring; fatigue detection; injury-related applications; and applications relevant to rehabilitation and return-to-sport. Reported model performance was summarized descriptively. Attention was given to the distinction between high apparent or internally validated performance and evidence derived from independent or external validation, as these represent different levels of evidentiary support. PROBAST+AI also emphasizes this distinction.

## 3. Results

### 3.1. Study Selection

A total of 393 records were identified across all searched databases using the applied search strategy (54 were retrieved from PubMed and 339 from Scopus; the search strategy is summarized in Table 1). Following removal of 58 duplicates, 335 records underwent title and abstract screening. A total of 305 records were excluded at this stage. Thirty reports were subsequently considered potentially eligible for full-text assessment. Following full-text assessment, 14 reports were excluded. Eight did not fulfil the predefined methodological or topical eligibility criteria, while six were excluded because they were conference papers or book chapters. A total of 16 peer-reviewed studies were therefore included in the final analysis.

### 3.2. Characteristics of Included Studies

The 16 included studies were published from 2017 [17] to 2026 [24,25,26,27]. Fourteen studies specifically investigated tennis [17,18,24,25,26,28,29,30,31,32,33,34,35,36], one specifically investigated padel [37], and one included tennis data as part of a broader multi-sport study [27]. The studies were characterized by considerable methodological heterogeneity. Wearable technologies included IMUs, accelerometers, gyroscopes, pressure sensors, and multimodal sensor configurations. Sensors were used to capture movement-related signals, technical execution, external load, and other variables associated with athletic performance. The principal characteristics of the included studies are summarized in Table 2, while detailed characteristics of the participants, wearable sensors and their placement, classification tasks, class distributions, validation strategies, and reported model-performance metrics for all included studies are provided in Supplementary Table S2.

### 3.3. Wearable Technologies and Extracted Data

Inertial sensing represented the most recurrent technological approach among the included studies. IMU-based systems enabled the acquisition of acceleration and angular-velocity data, which were subsequently processed to characterize player movement, technical actions, and skill-related characteristics [17,18,24,28,33,34,35,36]. Sensor placement varied according to the analytical objective. Wrist-worn devices were used for the recognition and classification of tennis activities and strokes [28,33], whereas bilateral IMU measurements were used to characterize player movements and discriminate skill level [25]. A cervical wearable system integrating a triaxial accelerometer, gyroscope, and magnetometer was also used for the recognition of tennis strokes [18]. These configurations illustrate the use of inertial signals at different anatomical locations to capture sport-specific movement patterns and technical execution.

Pressure-based sensing represented another relevant approach, particularly for applications involving grip characteristics and joint or movement states. Gao et al. [32] investigated a dual-mode pressure sensor combined with a deep-learning model for joint-state monitoring, whereas Zhang et al. [27] evaluated grip-pressure patterns using flexible piezoresistive pressure sensors and KNN classification. Pressure sensing therefore provided information that was complementary to inertial measurements, particularly for characterizing force- or pressure-related aspects of technical execution.

Several studies employed multimodal or distributed sensor configurations. Li and Wu [31] used a sensor array integrated into an intelligent insole, providing information related to lower-limb and foot-ground interactions. Yuan et al. [26] combined multimodal sensor information within a spatio-temporal graph neural-network framework for fatigue detection, integrating movement-related and physiological information. Han et al. [24] similarly employed a multisensor system incorporating biomechanical, physiological, and movement-related information for the analysis of injury patterns. These approaches indicate a broader transition from single-sensor movement recognition toward multimodal characterization of player status and external or internal load.

Overall, the included studies demonstrated considerable variability in sensor modality, anatomical placement, and configuration. Upper-limb placements, particularly at the wrist, were frequently used for technical-action and activity recognition, whereas other configurations, including cervical, bilateral, plantar/insole, and multimodal placements, were employed to capture different aspects of movement, loading, fatigue, or injury-related characteristics. Figure 2 provides a schematic overview of the principal sensor types and anatomical locations reported across the included studies.

### 3.4. Movement Recognition and Stroke Classification

A substantial proportion of the literature focused on the automated recognition and classification of tennis movements or technical actions. Pardo et al. [36] developed a wearable-sensor system for distinguishing different activities performed during tennis matches, while Ganser et al. [28] used a wrist-worn sensor system and deep neural networks to classify tennis shots. Similarly, Perri et al. [18] evaluated prototype ML algorithms based on a cervical wearable sensor incorporating a triaxial accelerometer, gyroscope, and magnetometer. Their algorithms achieved the highest accuracy for serves (98%) and groundstrokes (94%), whereas the detection of backhand slices and volleys was less accurate. Wu et al. [33] investigated sport-specific movement classification using a wrist-worn MEMS sensor in elite tennis players, whereas Gao and Zhang [34] used IMU and flexible resistive sensors combined with an improved SVM to recognize and evaluate tennis actions, reporting a mean accuracy of 95.35%. Chen [35] similarly investigated tennis-player pose recognition using IMU-derived data and a K-Nearest Neighbors (KNN) classifier.

### 3.5. Performance and Skill Assessment

The studies addressing performance and skill assessment investigated several distinct aspects of tennis performance, including player skill level, technical execution, expertise-related characteristics, and psychological or performance states.

#### 3.5.1. Skill-Level Classification

Aydin and Aydemir [25] developed a deep-learning framework based on bilateral IMU measurements to discriminate tennis players according to skill level. The model achieved an accuracy of 95.54%, indicating the potential of wearable-derived movement data to differentiate athletes with different levels of technical proficiency.

#### 3.5.2. Technical Execution and Expertise-Related Assessment

Gao and Zhang [34] investigated action recognition and evaluation in elite tennis players by combining IMU and flexible resistive sensor data with an improved support vector machine (SVM). Zhang et al. [27] focused on grip-pressure characteristics using flexible piezoresistive sensors to compare expert and amateur athletes. In the tennis component of their study, K-nearest neighbors (KNN) classification achieved 95.0% accuracy in the expert training condition compared with 84.6% in amateur players. These studies illustrate the use of wearable-derived signals to characterize technical execution and expertise-related differences.

#### 3.5.3. Psychological and Performance-State Assessment

Havlucu et al. [29] explored the possibility of detecting the psychological state referred to as being “in the zone” in elite tennis players using IMU data from a commercial smartwatch and coach-generated labels. An LSTM model achieved a test accuracy above 85%, suggesting the potential of wearable-derived data for identifying athlete states associated with performance.

### 3.6. Workload Monitoring

Several studies investigated the use of wearable-derived data to quantify external physical load during tennis. Whiteside et al. [17] applied inertial sensor data and machine learning to monitor hitting load, demonstrating the potential of sensor-derived information to characterize the external demands associated with different shot types. Similarly, Marutani et al. [30] applied Gaussian mixture modelling and linear discriminant analysis to acceleration-derived signals to monitor external physical load in tennis players. Their approach achieved 93% accuracy when using high-intensity-side mean and standard deviation parameters, compared with 63% when using the conventional overall mean. These studies indicate that wearable-derived acceleration data can be used to characterize aspects of the external physical demands associated with tennis play.

### 3.7. Fatigue Detection

Only one included study specifically investigated fatigue detection. Yuan et al. [26] developed a multimodal framework combining inertial measurement units (IMUs) and surface electromyography (sEMG) with a spatio-temporal graph convolutional network to classify fatigue during tennis serving. The study included 15 high-performance tennis players who performed a standardized serve-to-exhaustion protocol. The transition from the “fresh” to the “fatigued” state was operationalized using three predefined termination criteria: (1) a Borg Rating of Perceived Exertion (RPE) > 18; (2) peak ball velocity falling below 85% of the participant’s maximum recorded velocity for three consecutive serves; or (3) serve accuracy falling below 50% within a set. The protocol was terminated when the first of these criteria was reached. The proposed model achieved 95.2% accuracy and an F1 score of 0.94, with an inference latency of 8.4 ms. The study also identified fatigue-related changes in kinematic and neuromuscular characteristics, including reduced knee flexion and alterations in electromechanical delay.

### 3.8. Injury-Related Applications

#### 3.8.1. Injury-Related Prediction

Han et al. [24] developed a multimodal wearable-sensor framework to investigate spatiotemporal injury patterns in professional tennis players. The study incorporated LSTM networks, clustering, ensemble methods, and a Transformer-based model. The Transformer-based approach achieved 91.5% accuracy and an AUC of 0.956 for the reported injury-related prediction task. The authors identified the shoulder–elbow complex and lumbar–hip region as the predominant anatomical injury distributions. These findings indicate the potential of multimodal wearable-sensor data and ML approaches to characterize injury-related patterns; however, they should not be interpreted as evidence that wearable-based ML can prevent injuries.

#### 3.8.2. Injury-Related Biomechanics

No included study directly evaluated biomechanical variables as prospective predictors of injury occurrence. Several studies investigated movement, pose, or technical-action characteristics that may be relevant to biomechanical assessment, but these studies did not directly assess injury outcomes and were therefore considered within the corresponding movement-recognition or performance-related categories rather than as injury studies.

Overall, the evidence addressing injury-related applications remains limited. Importantly, no included study demonstrated the effectiveness of a wearable-sensor-based ML model for injury prevention, and prospective injury prediction remains insufficiently established in the current tennis and padel literature.

### 3.9. Padel-Specific Evidence

The evidence concerning padel was substantially more limited than that available for tennis. The only padel-specific study included in the present review was conducted by Cartes Domínguez et al. [37], who compared several ML and deep-learning approaches for padel shot classification, including dense neural networks, 1D-CNN, decision tree, KNN, SVM, and eigenvalue-based classification. The study demonstrated the feasibility of applying wearable-derived information to the automated classification of padel strokes.

### 3.10. Validation Strategies and Model Performance

A major methodological finding of this review was the heterogeneity in validation strategies. Cross-validation was commonly used, and several studies reported high classification or prediction performance. Whiteside et al. [17], for example, used 10-fold cross-validation, while Wu et al. [33] also reported a 10-fold cross-validation approach.

Importantly, however, genuinely subject-independent validation was considerably less common. Pardo et al. [36] explicitly aimed to develop a system robust to variability between subjects, and reported performance for seen and unseen subjects, with accuracy decreasing from 99.25% in seen subjects to 96.51% in an unseen participant. This represents one of the more informative validation approaches identified in the present review because it directly addresses generalization to a new athlete.

Conversely, several studies were based on repeated observations from a limited number of participants. Ganser et al. [28] analysed 5682 labelled shots from 16 players, whereas Yuan et al. [26] used data from 15 players for fatigue detection.

### 3.11. Risk of Bias

The adapted PROBAST+AI assessment identified substantial methodological concerns across the included literature, particularly regarding the evaluation of model performance (Supplementary Table S3). Most studies were judged to have a high overall risk of bias in model evaluation. The principal concerns related to the limited number of independent participants [18,24,25], the use of multiple stroke-level or sample-level observations from the same athletes [26], and the absence of clearly participant-independent validation procedures.

Several studies used k-fold cross-validation or random training/test splits at the recording or stroke level. Although these approaches provide repeated estimates of classification performance, they do not necessarily assess generalisation to previously unseen athletes when multiple observations from the same participant are distributed across training and evaluation datasets. This concern was particularly relevant to studies using large numbers of stroke recordings generated from relatively small participant samples.

Concerns were also identified regarding the transparency and completeness of model-development and validation procedures, including limited reporting of participant-level data separation, missing-data handling, preprocessing within resampling procedures, and independent external validation. In several studies, high classification accuracy was therefore obtained without sufficient evidence that the reported performance would generalise to athletes not represented in the training data.

One notable exception was the study by Yuan et al. [26], which used leave-one-subject-out cross-validation, ensuring that the athlete used for evaluation was not included in the corresponding model-development dataset. This design provided a more direct assessment of participant-independent generalisation.

Overall, the risk-of-bias assessment indicates that the current evidence base is characterised predominantly by exploratory or internally validated classification studies, with limited evidence from genuinely participant-independent or externally validated models. These findings should be considered when interpreting the apparently high classification accuracies reported across the literature.

The individual domain-level assessments are reported in Table 3, while an overview of the pathway from wearable-derived data to ML applications and validation strategies is provided in Figure 3.

In addition to participant selection and model validation, potential sources of bias related to sensor measurement were considered within the predictor/sensor domain. Reporting of sensor characteristics and acquisition procedures was heterogeneous across the included studies, particularly regarding sensor configuration, placement, calibration, sampling characteristics, and procedures for ensuring signal quality. Consequently, the potential influence of measurement-related factors such as motion artifacts, sensor misalignment, signal drift, measurement-range limitations, or synchronization between multiple sensor streams could not always be established from the available reports. These issues were therefore considered as potential sources of measurement uncertainty rather than assumed to represent demonstrated bias when sufficient evidence was not available.

## 4. Discussion

The present review summarizes the current evidence on the application of ML to wearable sensor data in tennis and padel. The main finding is that the literature is predominantly focused on movement and technical-action recognition, while performance-monitoring applications are increasingly represented. In contrast, evidence addressing fatigue, injury prevention, and particularly rehabilitation and return-to-sport remains limited. Overall, the available studies demonstrate the technical feasibility of combining wearable sensors with ML algorithms to extract meaningful information from racket-sport movements; however, substantial methodological heterogeneity and limited participant-independent validation currently restrict the translation of these approaches into routine clinical or sporting practice. Another relevant finding is the marked imbalance between tennis and padel research. Almost all included evidence concerned tennis, whereas only one study specifically investigated padel [37]. Thus, the evidence base across the two sports was substantially unbalanced, and the findings related to padel should be interpreted cautiously. The available padel study demonstrated the feasibility of ML-based classification of padel strokes using IMU-derived data, but the limited evidence prevents broader conclusions regarding generalizability or clinical applications. This represents an important research gap given the increasing popularity of padel and its specific biomechanical and tactical characteristics. Although tennis and padel share several technical demands, they differ in court dimensions, wall interactions, movement patterns, rally structure, and tactical requirements. Consequently, models developed and validated in tennis should not automatically be assumed to perform similarly in padel. Instead, the combined analysis provides a framework for identifying which methodological approaches appear common to racket-sport wearable applications and which areas require sport-specific validation.

Movement and stroke recognition represented the most extensively investigated application domain in the included studies. Across different sensor configurations, including IMUs, wrist-worn devices, pressure sensors, and multimodal systems, ML and deep-learning algorithms were able to classify tennis activities, technical strokes, poses, and movement patterns with generally high reported performance [17,18,28,32,33,34,35,36]. This finding is consistent with broader evidence that wearable sensors can provide continuous biomechanical information in real-world sporting environments, although their technical capabilities do not necessarily translate into demonstrated clinical utility for injury prevention and management [38,39,40,41]. The literature also indicates a progressive shift from simple activity recognition toward more complex applications. Several included studies investigated hitting load, external physical load, skill-level discrimination, action evaluation, and grip-pressure characteristics [24,27,30,34].

An additional consideration emerging from the present review concerns the substantial heterogeneity in wearable-sensor modality and anatomical placement. Inertial sensors, particularly IMUs, represented the most frequently used technology and were commonly positioned at the wrist or other body locations to capture acceleration and angular-velocity signals relevant to movement and technical-action recognition [17,18,24,28,33,34,35,36]. Pressure-based sensors provided complementary information on grip characteristics and joint or movement states [27,32], while insole-based and multimodal configurations enabled the integration of information from different anatomical regions and signal domains [24,26,33]. This heterogeneity is relevant when comparing ML performance across studies, because the information available to the model depends not only on the algorithm but also on the sensor modality, placement, sampling configuration, and characteristics of the acquired signal. Consequently, models developed using a specific sensor configuration should not necessarily be expected to generalize to different anatomical placements or multimodal settings. Greater standardization and more detailed reporting of sensor placement, orientation, sampling characteristics, preprocessing, and signal derivation would facilitate reproducibility and comparison across studies and may support the development of more generalizable wearable-based ML models.

One of the most important findings of this review concerns the validation of ML models. Although several studies reported very high accuracy or F1 scores, validation strategies were heterogeneous, and explicit evaluation in previously unseen or independent participants was less frequently reported [36,37]. Pardo et al. provide an illustrative example [36]. Although the model achieved 99.25% accuracy in seen subjects, performance decreased to 96.51% when evaluated on an unseen subject. This difference illustrates a fundamental issue in wearable-based ML: a model that performs well on observations from athletes represented in the training dataset may not necessarily demonstrate equivalent generalizability to new athletes. This concern is consistent with the broader literature on ML-based sports injury prediction. A recent systematic review and meta-analysis including 10 independent studies reported pooled sensitivity of 0.79 and specificity of 0.71 for ML-based sports-injury prediction models, while emphasizing substantial methodological differences and the potential influence of sample size, model type, validation strategy, and injury definition on model performance [42]. Similarly, systematic reviews of ML-based injury prediction have emphasized substantial heterogeneity in predictors, outcomes, modelling approaches, and validation procedures [12].

Compared with movement recognition and performance monitoring, applications addressing fatigue and injury prevention remain considerably less developed. One included study investigated fatigue detection during tennis serving using multimodal IMU and sEMG data and reported high classification performance [6]. This represents an interesting step toward real-time monitoring of physiological and biomechanical changes that may be relevant to training load and injury risk. Similarly, one recent included study investigated spatiotemporal injury patterns in professional tennis players using multimodal wearable data and ML approaches, reporting promising predictive performance [24]. However, these findings should be interpreted cautiously. Prediction of injury-related patterns is not equivalent to demonstrating that a model can prospectively prevent injuries. This distinction is consistent with the broader wearable-sensor literature. Preatoni et al. [9] concluded that, despite considerable interest in using wearable technologies for injury prevention and management, the evidence connecting wearable-derived biomechanical quantities with actual musculoskeletal injury and demonstrating readiness for implementation remained limited. Therefore, before wearable-based ML models can be considered reliable injury-prevention tools, future studies should include longitudinal cohorts, clearly defined injury outcomes, appropriate temporal prediction windows, independent validation, and comparison with clinically relevant baseline models. An important gap identified by the present review is the lack of substantial direct evidence for rehabilitation or return-to-sport applications in tennis and padel. Although rehabilitation is included within the scope of the present review, none of the included studies provides sufficient evidence to establish wearable-based ML as a validated tool for monitoring rehabilitation progression or determining readiness to return to sport. A recent systematic review of ML models for return-to-sport decisions after athletic injury identified an emerging body of evidence, but also highlighted substantial methodological heterogeneity in study design, predictive variables, model development, evaluation, and reporting [43]. The potential relevance of wearable sensors to rehabilitation is considerable. In principle, wearable-derived ML models could provide objective longitudinal measures of movement symmetry, technical execution, loading progression, and movement quality during recovery. However, these applications should not be extrapolated from movement-recognition studies alone. Rehabilitation research should involve injured athletes followed prospectively throughout recovery, incorporate clinically meaningful functional and patient-reported outcomes, and evaluate whether ML-derived information adds value beyond established clinical and return-to-sport assessments [44]. In this context, ML should be considered a decision-support tool rather than a replacement for clinical judgment. Another relevant finding is the marked imbalance between tennis and padel research. Almost all included evidence concerned tennis, whereas only one study specifically investigated padel [37]. The available padel study demonstrated the feasibility of ML-based classification of padel strokes using IMU-derived data, but the limited evidence prevents broader conclusions regarding generalizability or clinical applications. This represents an important research gap given the increasing popularity of padel and its specific biomechanical and tactical characteristics. Although tennis and padel share several technical demands, they differ in court dimensions, wall interactions, movement patterns, rally structure, and tactical requirements. Consequently, models developed and validated in tennis should not automatically be assumed to perform similarly in padel.

An additional methodological issue concerns the possibility of measurement bias originating at the wearable-sensor level. The performance of an ML model is inherently dependent on the quality and stability of the signals provided to it, and therefore high classification or prediction accuracy does not necessarily imply that the underlying sensor-derived measurements are accurate. IMU-based measurements may be affected by motion artifacts, sensor misalignment, soft-tissue effects, calibration errors, signal drift, and limitations in measurement range or sampling characteristics [32]. These limitations may become particularly relevant during high-intensity or impact-based movements. For example, recent work in a real tennis context demonstrated systematic measurement errors and saturation of an inertial sensor during high-intensity racket impacts, highlighting that sensor outputs may not always reproduce the magnitude of the mechanical stimulus with high fidelity [33]. Similarly, recent work on IMU-based position reconstruction has shown how sensor errors and drift can propagate through orientation estimation and numerical integration [34]. These considerations are important when interpreting ML performance, because errors or artifacts present in the input signals may be learned by the model and potentially affect its apparent performance and generalizability.

Multisensor configurations introduce additional methodological considerations. In the present review, Yuan et al. [26] combined IMUs with surface electromyography, while Han et al. [24] employed a multisensor wearable system for injury-related analysis. Although multimodal acquisition may provide complementary information across biomechanical and physiological domains, the use of multiple sensors may also increase the complexity of data acquisition and preprocessing. Potential sources of uncertainty include synchronization between sensor streams, differences in sampling rates or preprocessing pipelines, consistency of sensor placement and attachment, and possible interference with natural movement or participant comfort. These factors may be particularly relevant when signals from different modalities are integrated into a single ML model. However, because the included studies did not consistently report these aspects in sufficient detail, their actual contribution to bias could not be determined. Future studies should therefore report sensor synchronization procedures, acquisition settings, calibration, placement, and preprocessing in sufficient detail to facilitate reproducibility and assessment of measurement validity.

The main limitation of the available literature is its substantial methodological heterogeneity. The included studies differed considerably in participant characteristics, sensor type and placement, acquisition protocols, extracted variables, ML algorithms, outcome definitions, and validation strategies. Importantly, this heterogeneity also includes the quality and reporting of the underlying sensor measurements. Differences in sensor characteristics, acquisition settings, calibration, signal processing, and multimodal synchronization may affect the input data independently of the subsequent ML algorithm. This heterogeneity prevented meaningful quantitative pooling and justified the qualitative synthesis adopted in the present review. The number of independent participants was also generally limited in several studies. Importantly, a large number of recorded sensor observations or strokes does not necessarily compensate for a small number of independent athletes. In addition, incomplete reporting of participant-level data, sensor-measurement procedures, and validation strategies made direct comparison between studies difficult.

An additional limitation concerns the quality of the wearable-derived data used as input for ML models. Sensor placement, movement of the sensor relative to the underlying anatomical structures, skin and soft-tissue motion, motion artifacts, calibration errors, signal drift, and measurement-range limitations may introduce noise or systematic error into the acquired signals. When these factors are insufficiently controlled or reported, the data provided to the ML algorithm may contain measurement-related variability that is independent of the underlying movement or physiological phenomenon of interest. This may affect feature extraction and model development and may consequently influence both apparent performance and generalizability. Therefore, reported accuracy or F1 scores, including values above 95%, should not be interpreted in isolation as evidence of robust model validity. This is particularly relevant for studies presenting moderate or high risk of bias or limited participant-independent or external validation. High apparent performance may reflect characteristics of the specific dataset, participants, sensor configuration, or validation procedure and may not necessarily translate to comparable performance in previously unseen athletes or real-world settings.

Future research should move beyond proof-of-concept classification toward prospective, longitudinal, and externally validated applications. Larger multicentre datasets involving athletes different in ages, sexes, competitive levels, and playing styles would improve model generalizability [45]. Standardization of sensor placement, data acquisition, preprocessing, and reporting of ML performance would also facilitate comparison between studies. Attention should be given to validation in genuinely new populations. For injury prevention, studies should prospectively determine whether sensor-derived ML features can identify athletes at increased risk before injury occurs. For rehabilitation, longitudinal studies involving injured athletes should investigate whether wearable-derived ML measures can objectively quantify recovery and complement established return-to-sport criteria.

## 5. Conclusions

Overall, the present study indicates that ML applied to wearable sensor data provides accurate recognition and classification of tennis movements. The field is progressively moving toward more ambitious applications involving performance monitoring, fatigue detection, and injury-related assessment. However, the central challenge is no longer simply demonstrating that an algorithm can accurately classify sensor data; it is demonstrating that its predictions generalize to new athletes and provide information that influences sporting and clinical decision-making. Future studies should therefore combine rigorous sensor validation and standardized reporting with larger and more diverse cohorts, subject-independent and external validation, and prospective evaluation in realistic sporting and clinical settings.

If these methodological challenges are addressed, wearable sensor data combined with ML could become an important component of personalized training, injury prevention, rehabilitation, and return-to-sport pathways in tennis and padel.

## Figures and Tables

**Figure 1:**
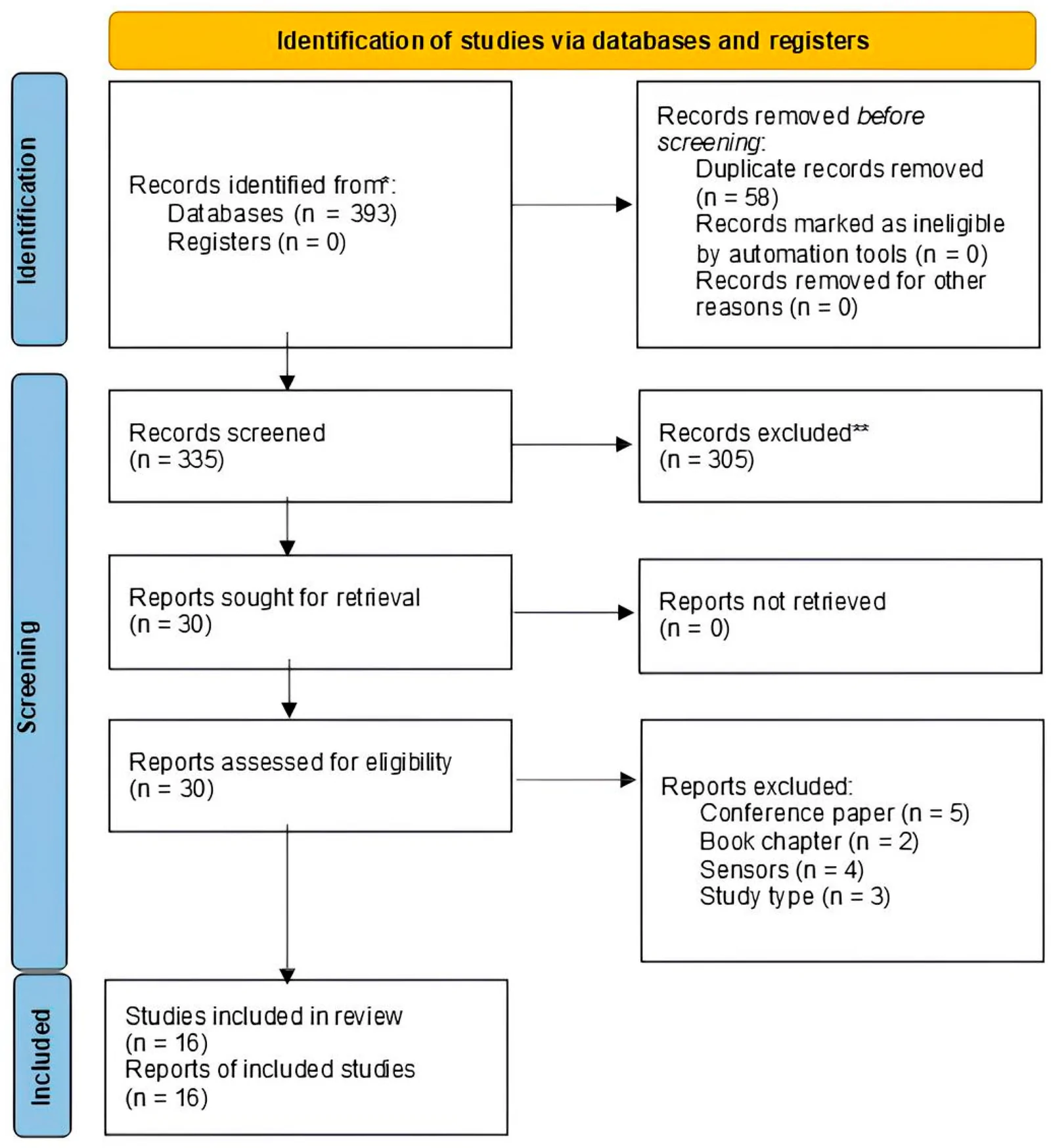
PRISMA flow diagram of the study selection process.

**Figure 2:**
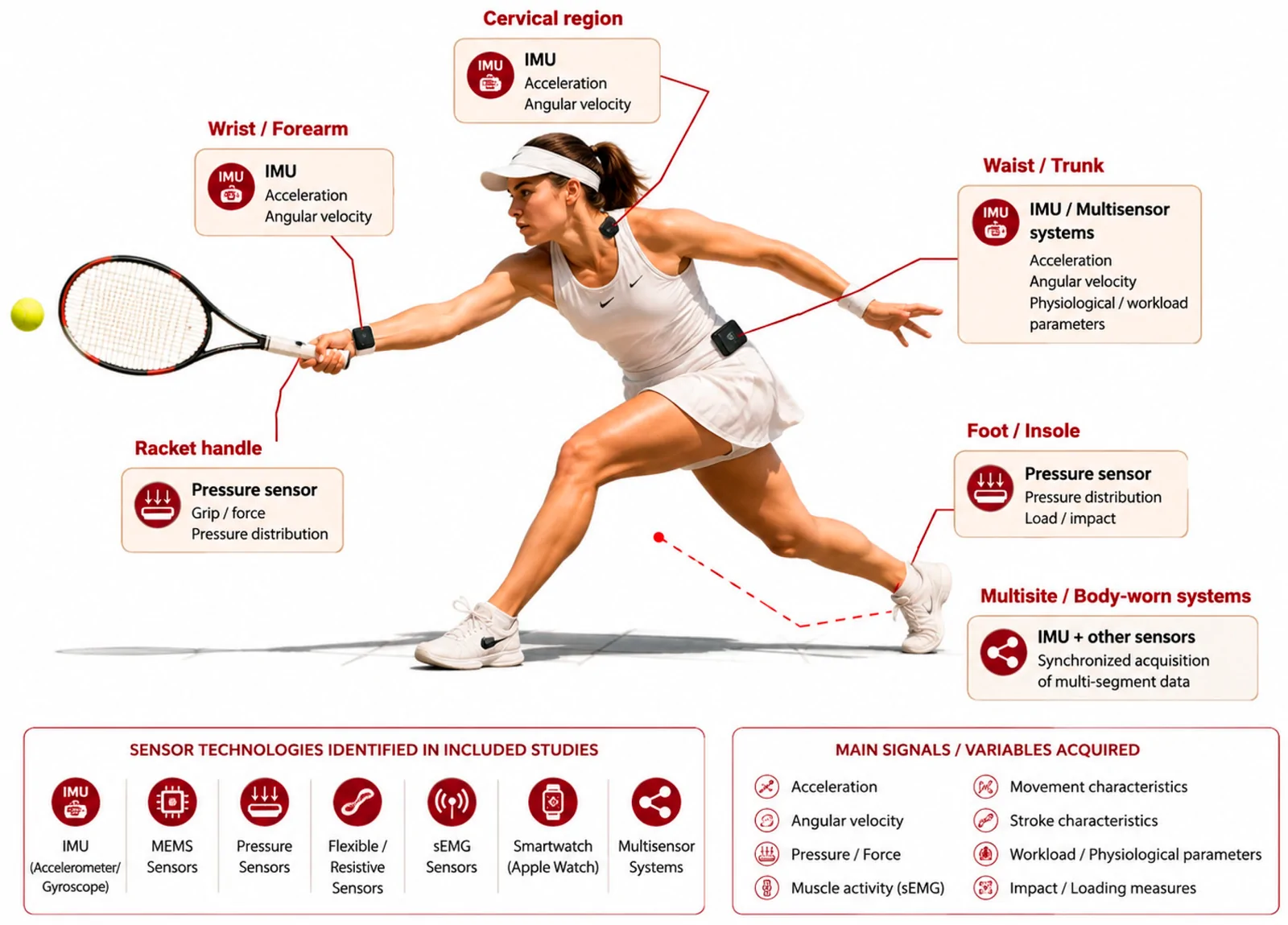
Wearable sensor technologies, anatomical placement, and extracted signals reported in tennis and padel. Schematic representation of the principal wearable sensor modalities identified across the included studies, their reported anatomical locations or configurations, and the main types of movement-related, biomechanical, physiological, or pressure-derived information used for machine-learning applications.

**Figure 3:**
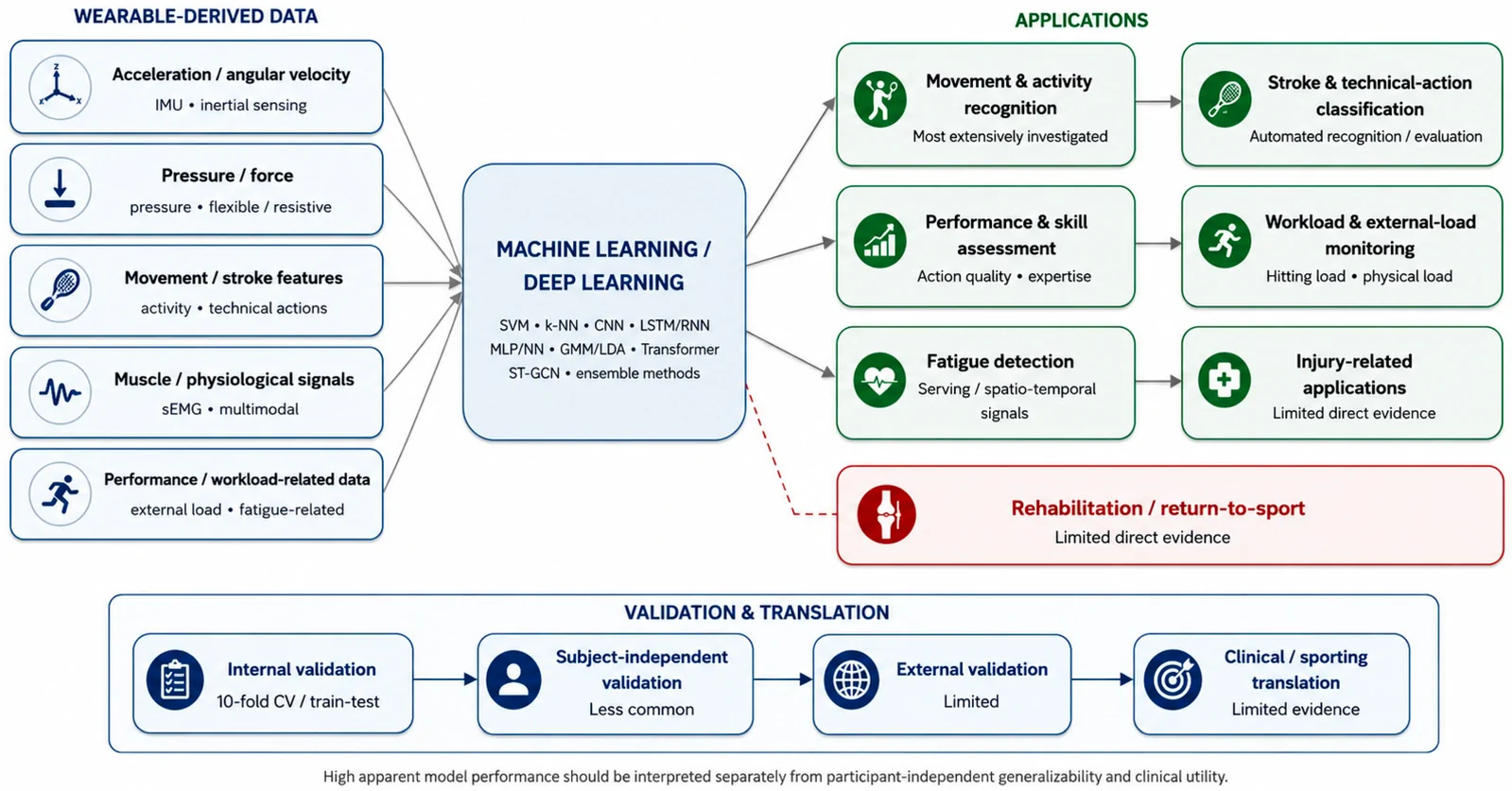
From wearable sensor data to machine-learning applications in tennis and padel. Overview of the main wearable-derived signals, machine-learning and deep-learning approaches, and applications identified across the included studies, ranging from movement and stroke recognition to perfor-mance assessment, workload and fatigue monitoring, and injury-related applications. The figure also highlights the limited evidence for rehabilitation and return-to-sport, and the heterogeneity of validation strategies.

**Table 1:** Search strategy.

PubMed
(tennis[Title/Abstract] OR padel[Title/Abstract] OR “racket sport*”[Title/Abstract] OR “racquet sport*”[Title/Abstract]) AND (wearable*[Title/Abstract] OR sensor*[Title/Abstract] OR accelerometer*[Title/Abstract] OR gyroscope*[Title/Abstract] OR “inertial measurement unit*”[Title/Abstract] OR IMU[Title/Abstract] OR GPS[Title/Abstract] OR smartwatch*[Title/Abstract] OR “wearable technolog*”[Title/Abstract]) AND ( “machine learning”[Title/Abstract] OR “deep learning”[Title/Abstract] OR “artificial intelligence”[Title/Abstract] OR classifier*[Title/Abstract] OR classification[Title/Abstract] OR prediction[Title/Abstract] OR predictive[Title/Abstract] OR “neural network*”[Title/Abstract] OR “support vector machine”[Title/Abstract] OR random forest[Title/Abstract])
Scopus
TITLE-ABS-KEY(tennis OR padel OR “racket sport*” OR “racquet sport*”) AND TITLE-ABS-KEY(wearable* OR sensor* OR accelerometer* OR gyroscope* OR “inertial measurement unit*” OR IMU OR GPS OR smartwatch* OR “wearable technolog*”) AND TITLE-ABS-KEY(“machine learning” OR “deep learning” OR “artificial intelligence” OR classifier* OR classification OR prediction OR predictive OR “neural network*” OR “support vector machine” OR “random forest”)

**Table 2:** Characteristics of the studies included in the systematic review.

First Author	Year	Country	Journal	Sport	Participants/Dataset	Wearable Sensor and Anatomical Placement	Application Domain
Whiteside et al. [17]	2017	Australia	International Journal of Sports Physiology and Performance	Tennis	19 athletes; 28,582 shots	Wrist	Performance monitoring
Perri et al. [18]	2022	Australia	Sensors	Tennis	Up to 8 high-performance players	Cervically mounted wearable	Movement/stroke recognition
Wu et al. [23]	2023	China	Proceedings of the Institution of Mechanical Engineers, Part P: Journal of Sports Engineering and Technology	Tennis	12 right-handed male elite athletes; 150 strokes each	Wrist-worn low-cost MEMS sensor	Movement/stroke recognition
Han et al. [24]	2026	China	Scientific Reports	Tennis	NR	Multisensor wearable system	Injury prevention/risk
Aydin & Aydemir [25]	2026	Türkiye	Sensors	Tennis	39 participants (11 elite, 28 amateur); 4,594 strokes incl. augmented samples	Bilateral IMUs	Performance monitoring
Yuan et al. [26]	2026	China; Republic of Korea	Frontiers in Bioengineering and Biotechnology	Tennis	15 high-performance players	IMUs + sEMG	Fatigue / injury prevention
Zhang et al. [27]	2024	China	Scientific Reports	Tennis + other sports	400 tennis strokes	Racket-handle flexible pressure sensor	Performance monitoring
Ganser et al. [28]	2021	Austria	Sensors	Tennis	16 players; 5,682 labelled shots	Wristband	Movement/stroke recognition
Havlucu et al. [29]	2022	Finland; Türkiye	Frontiers in Sports and Active Living	Tennis	4 professional players; 2 games; 2 elite coaches	Apple Watch Series 2	Performance monitoring
Marutani et al. [30]	2023	Japan	Frontiers in Physiology	Tennis	14 male collegiate + 55 male middle-aged players; 83 matches	Sportswear-type wearable sensor	Performance monitoring
Li & Wu [31]	2023	China	IEEE Sensors Journal	Tennis	NR	Intelligent insole sensor array	Movement/stroke recognition
Gao et al. [32]	2023	China	Journal of Sensors	Tennis	NR	Wearable flexible RCDM sensor	Movement/stroke recognition
Gao & Zhang [34]	2024	China	Systems and Soft Computing	Tennis	NR	IMU + flexible resistive sensors	Performance monitoring
Chen [35]	2024	China	International Journal of High Speed Electronics and Systems	Tennis	NR	IMU sensor	Movement/stroke recognition
Benages Pardo et al. [36]	2019	Spain	Sensors	Tennis	8 players (4F/4M)	Wrist + waist sensor nodes	Movement/stroke recognition
Cartes Domínguez et al. [37]	2023	Spain	Soft Computing	Padel	Dataset created for study; exact participant number NR	IMU device	Movement/stroke recognition

Abbreviations: F = female; M = male; NR = not reported; IMU = inertial measurement unit; RCDM = resistance/capacitance dual-mode; MEMS = micro-electromechanical systems; sEMG = surface electromyography.

**Table 3:** Machine learning approaches, validation strategies, reported performance, and methodological concerns.

First Author	ML Approach	Validation/Main Performance	Overall RoB	Applicability	Main Findings	Main Methodological Concern
Whiteside et al. [17]	6 ML models; cubic-kernel SVM	97.4% for 3 binned shot groups; 93.2% for 9 shot types; 10-fold CV	Some concerns	Some concerns	Wrist inertial data enabled accurate classification of shot types and supported automated monitoring of tennis hitting load.	10-fold CV reported; unclear subject-independent split/generalizability.
Perri et al. [18]	Prototype ML algorithms	Serve 98%; groundstrokes 94%; backhand slice 74%; volleys 41–44%; running classification 74%	Some concerns	High	A cervical wearable detected serves and groundstrokes accurately, but performance was lower for slices, volleys, and running actions.	Very small, selected cohort and prototype evaluation limit generalizability.
Wu et al. [23]	Machine-learning algorithms	Headline metric NR in accessible extraction; 10-fold CV reported	Some concerns	Some concerns	Wrist-worn MEMS data supported automated classification and real-time assessment of sport-specific movements in elite tennis training.	Homogeneous elite-male cohort; generalizability beyond the sample is limited.
Han et al. [24]	LSTM, clustering, ensemble methods, Transformer	Transformer prediction: 91.5% accuracy; AUC 0.956; shoulder-elbow 47.3%, lumbar-hip 31.8%	High	High	The multimodal framework identified shoulder-elbow and lumbar-hip regions as the predominant injury distributions and achieved high injury-related prediction performance.	Complex multimodal prediction framework; cohort/data provenance and true independent evaluation require careful appraisal.
Aydin & Aydemir [25]	CNN-BiLSTM; benchmark ML/DL models	95.54% accuracy	Some concerns	Some concerns	Bilateral IMU data and a CNN-BiLSTM model discriminated elite from amateur tennis strokes with high accuracy.	4,594 strokes include augmented samples; independent participant count is much smaller.
Yuan et al. [26]	ST-GCN; compared with LSTM, CNN, ST-TR	95.2% accuracy; F1 0.94; inference latency 8.4 ms	Some concerns	Some concerns	Multimodal spatio-temporal modelling accurately detected fatigue during tennis serving and supported low-latency inference.	15 players; repeated measurements and external validation not established from accessible report.
Zhang et al. [27]	KNN	KNN accuracy: 95.0% training (expert) vs 84.6% amateur in reported conditions	Some concerns	Some concerns	Racket-handle pressure patterns distinguished expert and amateur tennis performance, with higher classification accuracy in experts.	Participant-level sample and independence of training/testing require attention.
Ganser et al. [28]	FCN and ResNet; SVM trigger	F1 96% main shots; F1 94% expanded set	Some concerns	Some concerns	Deep neural networks applied to wristband data achieved high F1 scores for automated recognition of major and expanded tennis-shot categories.	Repeated shots from 16 players; subject independence of validation should be considered.
Havlucu et al. [29]	Deep recurrent neural network/LSTM	Above 85% test accuracy	High	High	Smartwatch-derived IMU data showed proof-of-concept potential for identifying the coach-labelled psychological state of being ‘in the zone’.	Very small cohort, limited games, and subjective coach-based labeling; preliminary proof-of-concept.
Marutani et al. [30]	Gaussian mixture model + linear discriminant analysis	93% accuracy using high-intensity-side mean/SD vs 63% conventional overall mean	Some concerns	Some concerns	High-intensity acceleration features substantially improved classification of external physical load compared with the conventional overall-mean approach.	Selected male samples; classification rather than prospective prediction; external generalizability limited.
Li & Wu [31]	Dual-model CNN	Exact performance metric NR in accessible extraction	Some concerns	High	An intelligent insole sensor network combined with a dual-model CNN demonstrated the feasibility of wearable-based recognition and analysis of tennis movements.	Participant count and validation details were not sufficiently available in the accessible extraction; confirm from full text.
Gao et al. [32]	LSTM/RNN	97.21% combined resistance+capacitance; resistance 74%; capacitance 76.93%; 4:1 split	High	High	Combining resistance and capacitance signals markedly improved tennis joint-state recognition over either sensing modality alone.	Participant dataset unclear; 4:1 split may allow dependence between training and testing observations.
Gao & Zhang [34]	Improved SVM	Mean accuracy 95.35%; forehand 96.38%	Some concerns	Some concerns	Combined IMU and flexible-resistive sensor data enabled accurate recognition and evaluation of tennis actions, with the best result for forehand strokes.	High reported accuracy, but sample size and independence of test data require verification.
Chen [35]	K-nearest neighbors	Exact performance metric NR in accessible extraction	Some concerns	High	IMU-derived features combined with KNN supported automated recognition of tennis-player poses.	Accessible extraction does not provide enough detail on sample size and independent validation.
Benages Pardo et al. [36]	Convolutional autoencoder + MLP; semi-supervised deep learning	99.25% for seen subjects; 96.51% for unseen subject	Some concerns	High	Wearable data accurately distinguished tennis activities, with only a modest decrease in accuracy for an unseen participant.	Small participant cohort; an unseen-subject result is reported, but precision/generalizability remain limited.

Abbreviations: RoB, risk of bias; ML, machine learning; CV, cross-validation; SVM, support vector machine; CNN, convolutional neural network; LSTM, long short-term memory; KNN, k-nearest neighbors; IMU, inertial measurement unit.

## Data Availability

The original contributions presented in this study are included in the article. Further inquiries can be directed to the corresponding authors.

## Supplementary Materials

The following supporting information can be downloaded at: https://www.mdpi.com/article/10.3390/jfmk11040391/s1. Supplementary Table S1. Database search details and complete search strings. Supplementary Table S2. Characteristics of included studies, participants, sensors, classification tasks, and validation strategies. Supplementary Table S3. Adapted PROBAST+AI assessment tool for machine-learning studies using wearable sensor data in tennis and padel. Supplementary Table S4. Risk-of-bias assessment. Supplementary File S1. PRISMA 2020 Checklist. Ref [46] is cited in the supplementary materials.
