# Supplementary material for: Machine Learning Applications for Wearable Sensor Data in Tennis and Padel: A Systematic Review of Movement Recognition, Performance Monitoring, and Emerging Injury-Related Applications
Source: J Funct Morphol Kinesiol. 2026 Sep 29;11(4):391. doi: 10.3390/jfmk11040391 (PMC13692505; doi:10.3390/jfmk11040391)
Supplement: Supplementary file 1 [file jfmk-11-00391-s001.zip › jfmk-4574577-Supplementary Tables.pdf]

Supplementary Table S1. Database search details and complete search strings.

Database	Search date	Search period	Complete search string
**PubMed**	August 20, 2026	Database inception to August 1, 2026	(tennis[Title/Abstract] OR padel[Title/Abstract] OR "racket sport*"[Title/Abstract] OR "racquet sport*"[Title/Abstract]) AND (wearable*[Title/Abstract] OR sensor*[Title/Abstract] OR accelerometer*[Title/Abstract] OR gyroscope*[Title/Abstract] OR "inertial measurement unit*"[Title/Abstract] OR IMU[Title/Abstract] OR GPS[Title/Abstract] OR smartwatch*[Title/Abstract] OR "wearable technolog*"[Title/Abstract]) AND ("machine learning"[Title/Abstract] OR "deep learning"[Title/Abstract] OR "artificial intelligence"[Title/Abstract] OR classifier*[Title/Abstract] OR classification[Title/Abstract] OR prediction[Title/Abstract] OR predictive[Title/Abstract] OR "neural network*"[Title/Abstract] OR "support vector machine"[Title/Abstract] OR random forest[Title/Abstract])
**Scopus**	August 20, 2026	Database inception to August 1, 2026	TITLE-ABS-KEY(tennis OR padel OR "racket sport*" OR "racquet sport*") AND TITLE-ABS-KEY(wearable* OR sensor* OR accelerometer* OR gyroscope* OR "inertial measurement unit*" OR IMU OR GPS OR smartwatch* OR "wearable technolog*") AND TITLE-ABS-KEY("machine learning" OR "deep learning" OR "artificial intelligence" OR classifier* OR classification OR prediction OR predictive OR "neural network*" OR "support vector machine" OR "random forest")

Supplementary Table S2. Characteristics of included studies, participants, sensors, classification tasks, and validation strategies.

Study	Independent participants	Age / sex	Sport level / health status	Sensor and placement	Classification task / no. classes	Class distribution	Validation strategy	Accuracy	Sensitivity / specificity
Whiteside et al., 2017	19 (8F, 11M)	NR; 8F/11M	Elite tennis athletes; health status NR	IMU; wrist	Shot classification: 3 binned classes (overhead, forehand, backhand); 9 original shot types	28,582 shots; exact class distribution NR	10-fold cross-validation	97.4% (3 binned classes); 93.2% (9 shot types)	NR / NR
Benages Pardo et al., 2019	8 (4F, 4M)	19–30 yr; 4F/4M	Player characteristics reported; competitive level/health status NR	2 SensorTags; wrist + waist	Tennis vs non-tennis; 7 normal-activity classes; tennis-stroke classifier: 5 outputs (4 strokes + rejection)	Training/test distributions reported; stroke TEST2: 1509 FH, 1508 BH, 1520 volley; 1495 lob	TEST1: held-out data from same players; TEST2: all data from player 1, unseen during training; 10 cross-validation runs used for architecture selection	>96.5% unseen-player stroke accuracy; 99.25% training accuracy	Sensitivity/specificity NR
Ganser et al., 2021	16	13–70 yr; sex distribution NR	Predominantly amateur; health status NR	Wristband with 11 signals including IMU; wrist	3 main shot classes; 5-class expanded classification	5682 labelled shots; backhand slice 6.35%, forehand slice 2.87%; detailed distribution reported in source	Stratified K-fold validation repeated over 50 iterations; exact K not specified in accessible text	NR	F1 = 96% (3 classes), 94% (5 classes); sensitivity/specificity NR
Perri et al., 2022	8 stroke-validation players; 2 movement-validation participants	Stroke: 15.5 ± 1.6 yr, all male; movement: 30 and 37 yr, male	Junior-elite/high-performance; movement participants healthy; stroke-group health status NR	Triaxial accelerometer + gyroscope + magnetometer; cervical pouch between scapulae	Stroke: 3 classes (forehand, backhand, serve); movement: 4 algorithm-defined classes	NR	Validation against manual coding/reference labels; no conventional cross-validation reported	Serve 98%; forehand 94%; backhand 94%	NR / NR
Havlucu et al., 2022	4 professional players	NR; players male	Professional; health status NR	Apple Watch Series 2; wrist	Binary: in-the-zone vs out-of-the-zone	Approximately 50:50 labels	Random train/test splitting repeated 5 times; no participant-independent split reported in this procedure	≈85% test accuracy	NR / NR
Marutani et al., 2023	69 (14 collegiate; 55 middle-aged)	19.5 ± 1.4 and 54.8 ± 8.7 yr; all male	Collegiate and middle-aged players in national championship qualifier rounds; health status NR	Inertial sensor integrated in sportswear; upper back	2 groups: collegiate vs middle-aged	14 vs 55 participants	Linear discriminant analysis; no cross-validation procedure reported	93% proposed GMM-derived features; 63% conventional feature	NR / NR
Li & Wu, 2023	NR	NR	NR	Intelligent insole sensor array; foot/insole	Tennis gait/trajectory recognition; classes NR	NR	NR	NR; trajectory error <6% reported	NR / NR
Cartes Domínguez et al., 2023	12	NR; mixed gender	Beginner to professional levels represented; health status NR	6-DOF IMU; wrist	13 padel-stroke classes	Class counts not fully summarized; exact distribution NR	Training/test evaluation after hyperparameterisation; participant-independent validation NR	93.35% maximum accuracy (1D-CNN, temporal-series input)	Precision/recall/F1 reported; sensitivity/specificity NR
Gao et al., 2023	NR	NR	NR	RCDM flexible wearable sensor; placement NR	Tennis movement-state recognition; classes NR	NR	NR	97.21% recognition rate	NR / NR

Study	Independent participants	Age / sex	Sport level / health status	Sensor and placement	Classification task / no. classes	Class distribution	Validation strategy	Accuracy	Sensitivity / specificity
Wu et al., 2023	12	NR; all male	Elite; right-handed	Single low-cost MEMS sensor; wrist-worn	Stroke classification; exact class structure NR	NR	10-fold cross-validation during training	99.0% highest test accuracy	NR / NR
Gao & Zhang, 2024	NR	NR	Elite, sub-elite, and amateur groups	IMU + flexible resistive sensors; placement NR	Action recognition across player-level groups; exact number of classes NR	Three skill groups; exact sample distribution NR	NR	95.35% elite; 97.67% sub-elite; 94.08% amateur	NR / NR
Chen, 2024	NR	NR	NR	IMU; placement NR	Tennis-player pose recognition; classes NR	NR	NR	NR	NR / NR
Han et al., 2026	45	19–32 yr; 24M/21F	Professional; ATP/WTA ranking 50–250; injury events monitored prospectively	Bilateral wrist IMUs; dominant shoulder IMU; lumbar IMU; left-forearm PPG; cervical GPS; bilateral force-sensing insoles; temperature/barometric sensors	Injury-risk/pattern prediction; exact class structure NR	NR	Sliding-window/time-aware validation reported; exact participant-independent split NR	91.5% Transformer accuracy; AUC 0.956	NR / NR
Aydin & Aydemir, 2026	39 (11 elite, 28 amateur)	Age/sex NR in verified abstract	Elite and amateur; relevant health/injury status should be taken from full text if retained	Bilateral wrist IMUs	Expertise/stroke classification; four stroke types analysed; exact class structure NR	4594 strokes including augmented samples; exact class distribution NR	Cross-validation procedure: retain only if confirmed from full text; otherwise NR	95.54%	Sensitivity/specificity NR
Yuan et al., 2026	15	21.4 ± 2.3 yr; 10M/5F	Semi-professional/high-performance collegiate; ITN ≤4; no musculoskeletal injury in previous 6 months	8 IMUs (foot, shank, thigh, pelvis, trunk, upper arm, forearm, hand) + 6 sEMG sensors	2 classes: fresh vs fatigued	NR	Leave-one-subject-out cross-validation	95.2 ± 2.1%	95.5% sensitivity / 94.5% specificity
Zhang et al., 2024	NR	NR	Expert vs amateur; health status NR	Flexible piezoresistive pressure sensor; racket handle	Expert vs amateur performance/skill discrimination; 2 groups	NR	Training-set KNN classification; independent test validation NR	95.0% expert; 84.6% amateur (training accuracy)	NR / NR

Abbreviations: NR, not reported; IMU, inertial measurement unit; RCDM, resistance/capacitance dual-mode; sEMG, surface electromyography; LDA, linear discriminant analysis; CNN, convolutional neural network.
Note: Participant numbers refer to independent athletes whenever reported and are distinguished from recorded strokes, samples, or augmented observations. Performance metrics are reported using the terminology of the original publication; F1 score, precision, recall, recognition rate, AUC, and training accuracy are not re-labelled as accuracy, sensitivity, or specificity. NR = not reported.

Supplementary Table S3. Adapted PROBAST+AI assessment tool for machine-learning studies using wearable sensor data in tennis and padel.

Phase	Domain	ID	Adapted signalling question
**Model development**	Participants & data sources	1.1	Were the data sources appropriate for developing the reported ML classification/prediction model?
		1.2	Was the study design appropriate for development of the reported ML model?
		1.3	Did the inclusion and exclusion of participants/data result in a dataset reasonably representative of the population and use scenario targeted by the model?
	Predictors	2.1	Were wearable-sensor predictors/signals defined, acquired and processed consistently across participants or recordings?
		2.2	Was preprocessing of wearable-sensor data performed consistently across participants and datasets?
		2.3	Were predictor measurements obtained without knowledge of the target classification/outcome in a way that could introduce bias?
		2.4	Were all predictors available at the intended time of model deployment/use?
	Outcomes	3.1	Was the target outcome/class label defined and determined appropriately?
		3.2	Was the target outcome/class label determined consistently across participants and recordings?
		3.3	Was outcome/label determination performed independently of predictor information, where relevant?
		3.4	Where temporal prediction was involved, was the interval between predictor acquisition and outcome determination appropriate?
	Analysis	4.1	Was the amount of independent participant/data information adequate for the complexity of the developed model?
		4.2	Were continuous/categorical inputs and derived sensor features handled appropriately?
		4.3	Were missing, incomplete or censored sensor recordings handled appropriately?
		4.4	If class-imbalance methods were used, was model performance subsequently evaluated using data that were not artificially corrected for imbalance?
		4.5	If training/test data were separated, was data leakage between development and evaluation datasets adequately prevented?
		4.6	If resampling/cross-validation was used, were all relevant model-development steps repeated within each resampling iteration?
		4.7	Were model performance measures appropriate for the classification/prediction task and reported adequately?
**Model evaluation**	Participants & data sources	1.1	Were the data used for model evaluation appropriate for assessing the reported ML model?
		1.2	Was the evaluation design appropriate for assessing model performance?

Supplementary Table S3. Adapted PROBAST+AI assessment tool for machine-learning studies using wearable sensor data in tennis and padel.

Phase	Domain	ID	Adapted signalling question
		1.3	Were the participants/recordings used for evaluation representative of the intended target population or use setting?
	Predictors	2.1	Were wearable-sensor predictors defined and assessed consistently across the evaluation data?
		2.2	Was preprocessing of predictors applied consistently to evaluation data?
		2.3	Were predictor measurements obtained without information that would not be available at intended model use?
		2.4	Were the predictors used for evaluation available at the intended time of model deployment?
	Outcomes	3.1	Was the target outcome/class label determined appropriately in the evaluation dataset?
		3.2	Was outcome determination consistent across participants/recordings?
		3.3	Was outcome determination independent of predictor information where relevant?
		3.4	Where temporal prediction was involved, was the interval between predictor and outcome assessment appropriate?
	Analysis	4.1	Was evaluation based on more than apparent/in-sample performance?
		4.2	Was the amount of independent participant/data information adequate for evaluation of model performance?
		4.3	Were missing or incomplete recordings handled appropriately in the evaluation?
		4.4	If class imbalance was corrected during development, was evaluation performed on data without artificial class correction?
		4.5	If training/test splitting was used, was information leakage between training and test data avoided?
		4.6	If resampling was used for evaluation, were all relevant development steps repeated within each resampling iteration?
		4.7	Were appropriate performance measures used to evaluate the classification/prediction model?

Supplementary Table S4. Risk-of-bias assessment.

Study	Model/task evaluated	Evaluation strategy	Participants/data sources	Predictors	Outcome	Analysis	Overall RoB	Main rationale
Whiteside et al., 2017	Stroke classification from wrist IMU	10-fold cross-validation	High	Low	Low	High	High	19 athletes and 28,582 shots; performance was evaluated by 10-fold CV, but participant-independent separation was not established, creating potential dependence/leakage between training and evaluation samples. (PubMed)
Benages Pardo et al., 2019	Tennis activity/stroke classification	Training/test classification	High	Low	Low	High	High	Only 8 participants; although training and test performance were reported, the available report does not establish robust participant-independent external validation. (PubMed Central (PMC))
Ganser et al., 2021	Neural-network stroke classification	Stratified K-fold validation	High	Low	Low	High	High	16 players and 5,682 labelled shots; stratified K-fold was performed at shot level, with potential correlation between samples from the same player across folds. (DOI)
Perri et al., 2022	Stroke and movement detection	Comparison with manual coding	High	Unclear	Low	High	High	Validation involved only eight junior-elite players for stroke data and two additional participants for movement validation; the proprietary classifier limited transparency of model development and independent generalisation. (Opus UTS)
Havlucu et al., 2022	"Zone"/optimal-performance classification	Five train-test splits	High	Low	Unclear	High	High	Only four professional players; personalised models performed well, whereas cross-player generalisation was close to chance, demonstrating limited generalisability. (PubMed Central (PMC))

Supplementary Table S4. Risk-of-bias assessment.

Study	Model/task evaluated	Evaluation strategy	Participants/data sources	Predictors	Outcome	Analysis	Overall RoB	Main rationale
Marutani et al., 2023	Player-group classification based on acceleration-derived features	LDA classification	Low	Low	Low	High	High	Relatively large participant sample (14 collegiate + 55 middle-aged players), but classification performance was not based on a clearly independent participant-level validation dataset; the reported approach is primarily exploratory. (Frontiers)
Li & Wu, 2023	Gait/trajectory recognition using DMCNN	Model-based classification/trajectory assessment	Unclear	Unclear	Unclear	Unclear	Unclear	The accessible publication information confirms the DMCNN and intelligent-insole framework but provides insufficient detail to reconstruct participant-level model evaluation and leakage control. (ResearchGate)
Cartes Domínguez et al., 2023	13-class padel-stroke classification	64% training / 16% validation / 20% test	High	Low	Low	High	High	Dataset contained 12 players and >2,000 strokes; random data partitioning was reported, but participant-independent testing was not established. Extensive hyperparameter tuning further increases the importance of strict separation of development and test data. (Springer Nature Link)
Gao et al., 2023	Joint-posture classification using RCDM sensor/LSTM	4:1 training/test split	High	Low	Low	High	High	The model was evaluated on a training/test split, but the publication does not establish independent participant-level validation; the task involved multiple posture samples from the same experimental subjects. (Wiley Online Library)

Supplementary Table S4. Risk-of-bias assessment.

Study	Model/task evaluated	Evaluation strategy	Participants/data sources	Predictors	Outcome	Analysis	Overall RoB	Main rationale
Wu et al., 2023	Tennis-stroke classification using wrist MEMS	10-fold cross-validation	High	Low	Low	High	High	12 elite athletes and 150 strokes each; 10-fold CV was used, but participant-independent evaluation was not demonstrated, allowing potential within-athlete dependence across folds. (Sage Journals)
Gao & Zhang, 2024	Tennis-action/skill-level classification using IMU + flexible resistive sensors	Classification evaluation	High/Unclear	Low	Low	High	High	The study reports an improved SVM and high classification accuracy, but the available report does not provide sufficient detail to establish independent participant-level validation and leakage control. (DOAJ)
Chen, 2024	Tennis posture recognition using IMU + KNN	Classification evaluation	Unclear	Low	Low	High/Unclear	High	The study reports calibrated IMU data, filtering, feature extraction and KNN classification, but participant numbers and independent validation procedures are insufficiently reported in accessible material. (ResearchGate)
Han et al., 2026	Injury-pattern/risk prediction from multisensor data	Temporal cross-validation / train-validation-test framework	Unclear	Low	Low	Low–Unclear	Unclear	The study reports temporally ordered training/validation/testing, 5-fold temporal cross-validation, and leakage prevention; however, the participant/data-source structure and independence of injury observations require caution when interpreting model performance. (Nature)
Aydin & Aydemir, 2026	Expertise/stroke classification from bilateral IMUs	5-fold stratified cross-validation	High	Low	Low	High	High	39 participants but 4,594 stroke samples; stratified CV preserves class ratios but does not establish participant-independent testing, so repeated samples

Supplementary Table S4. Risk-of-bias assessment.

Study	Model/task evaluated	Evaluation strategy	Participants/data sources	Predictors	Outcome	Analysis	Overall RoB	Main rationale
								from individuals can contribute to both training and evaluation. (PubMed Central (PMC))
Yuan et al., 2026	Fatigue-state classification using IMU+sEMG	Leave-one-subject-out CV	Low	Low	Low	Low	Low	15 high-performance players; LOSO validation explicitly tests each athlete as unseen data, with training/validation confined to the remaining subjects and standard accuracy, precision, recall and F1 reporting. (Frontiers)
Zhang et al., 2024	Tennis grip-pressure classification	70% training / 30% test	High	Low	Low	High	High	Only two tennis athletes (one expert, one amateur); the 400-stroke dataset was randomly split into training/test samples, so repeated observations from the same individuals appear in both sets and participant-level generalisation cannot be established. (Nature)
